# The Validity and Reliability of a Kinect v2-Based Gait Analysis System for Children with Cerebral Palsy

**DOI:** 10.3390/s19071660

**Published:** 2019-04-07

**Authors:** Yunru Ma, Kumar Mithraratne, Nichola C. Wilson, Xiangbin Wang, Ye Ma, Yanxin Zhang

**Affiliations:** 1 Department of Exercise Sciences, Faculty of Science, University of Auckland, Auckland 1023, New Zealand; y.ma@auckland.ac.nz; 2 Auckland Bioengineering Institute, University of Auckland, Auckland 1010, New Zealand; p.mithraratne@auckland.ac.nz; 3 Department of Surgery, School of Medicine, Faculty of Medical and Health Science, University of Auckland, Auckland 1023, New Zealand; n.wilson@auckland.ac.nz; 4 College of Rehabilitation Medicine, Fujian University of TCM, Fuzhou 350122, China; wangxbin@fjtcm.edu.cn; 5 Research Academy of Grand Health, Faculty of Sports Science, Ningbo University, Ningbo 315000, China; maye@nbu.edu.cn

**Keywords:** Kinect, cerebral palsy, gait analysis, reliability, validity, kinematics

## Abstract

The aim of this study is to evaluate if Kinect is a valid and reliable clinical gait analysis tool for children with cerebral palsy (CP), and whether linear regression and long short-term memory (LSTM) recurrent neural network methods can improve its performance. A gait analysis was conducted on ten children with CP, on two occasions. Lower limb joint kinematics computed from the Kinect and a traditional marker-based Motion Analysis system were investigated by calculating the root mean square errors (RMSE), the coefficients of multiple correlation (CMC), and the intra-class correlation coefficients (ICC*_2,k_*). Results showed that the Kinect-based kinematics had an overall modest to poor correlation (CMC—less than 0.001 to 0.70) and an angle pattern similarity with Motion Analysis. After the calibration, RMSE on every degree of freedom decreased. The two calibration methods indicated similar levels of improvement in hip sagittal (CMC—0.81 ± 0.10 vs. 0.75 ± 0.22)/frontal (CMC—0.41 ± 0.35 vs. 0.42 ± 0.37) and knee sagittal kinematics (CMC—0.85±0.07 vs. 0.87 ± 0.12). The hip sagittal (CMC—0.97±0.05) and knee sagittal (CMC—0.88 ± 0.12) angle patterns showed a very good agreement over two days. Modest to excellent reliability (ICC*_2,k_*—0.45 to 0.93) for most parameters renders it feasible for observing ongoing changes in gait kinematics.

## 1. Introduction

Three-dimensional gait analysis (3DGA) is a useful tool for providing quantitative information for treatment decision making and outcome assessment, especially for children with cerebral palsy (CP) [1,2]. However, high financial burden for equipment purchasing and maintenance, technical expertise requirements for experimental operation, and data processing, limit its clinical applications. Due to its marker-based optoelectronic tracking strategies, participants might become conscious of being observed which might lead to over-performance, rather than their natural daily gait [3]. Moreover, accurately placing markers is a challenge for children who are restless and might be less compliant. The shortcomings of traditional 3DGA systems necessitate the development of cost-effective and user-friendly motion capture tools for clinical practice, in particular for lesser developed areas, community health service centers, and home-based observation. 

With the advances in depth-imaging techniques, the Kinect released by Microsoft is able to deliver full-body three dimensional (3D) information in real-time [4]. The structured 3D light scanner consists of an RGB camera and a depth sensor, which can collectively capture 3D motion, as well as track joint movements, without any markers or handheld controllers, under any lighting conditions [5]. According to the systematic skeletal system adopted by the Kinect SDK, joint centers that root from hip joints and extend to the head and extremities, can be built without any calibration process [5,6,7]. Based on its imaging characteristics and relatively low cost (usually around 200 USD), the Kinect has the potential to be applied as a portable and easy-to-operate markerless motion capture system, with advantages in its uncomplicated calibration and setup process. 

To date, several studies have investigated the feasibility of applying different versions of the Kinect sensor to human movement capture, under varying circumstances. For instance, the Kinect was reported to provide valid and reliable results in posture control and balance assessment, especially in gross motor tasks [8,9,10]. Some ergonomic studies have proved that the Kinect is reliable and highly valid in shoulder angle estimations, when placed in front of subjects in working conditions [11,12]. Studies in the observation of Kinect-based lower limb joint kinematics show inconsistent results of reliability and validity, for some sport-related movement tasks, such as squats, drop jumps, and side-cuts [13,14,15,16]. 

With regards to gait analysis, a recent literature review summarized the results of twelve studies and concluded that the Kinect had good validity for several spatiotemporal parameters, and a poor validity in kinematic parameters [17]. Only one study assessed the reliability of the Kinect during gait. It reported that most of the kinematic variables had poor to modest reliability, except for the peak knee flexion angle during the swing phase [18]. However, three studies showed that, although measurement errors exist, the joint angles measured by the Kinect follow the trend of the traditional 3D motion capture recordings, especially the hip and knee angles in the sagittal plane [19,20,21]. The joint angles calculated by the Kinect were highly correlated with the traditional marker-based motion capture system during 20%–60% of the gait cycle [21]. Xu et al. found that the Kinect kinematic measurement errors are approximately proportional to the magnitude of the calculated joint angles, and so the measurement errors can be reduced through a linear regression calibration method [12]. 

Moreover, in order to improve the tracking accuracy of the Kinect, Timmi et al. developed a novel tracking method that employed custom-designed marker clusters and computer vision techniques. It demonstrated good agreement with maker-based motion capture system, over a range of overground gait speeds [22]. Both the parallel translation and linear regression methods can be deemed as simplified calibration methods that probably decrease the differences between the Kinect and its reference motion capture system. Meanwhile, joint angles during gait are contextually related and periodic from the time-domain sequences [23]. The recurrent neural network method, which can compute a sequence of outputs when given sequential inputs [24], has shown promising improvements in the Kinect skeletal-based action detection and recognition [25,26]. 

The Kinect-based joint angle trajectories followed the trend of its referential counterpart, and the measurement errors were in stable magnitudes [19,21]. Moreover, the Kinect had the ability of accurately assessing spatiotemporal parameters during gait, which implied that the Kinect had the potential to be utilized as a low-cost device for gait assessment [18,19,27]. However, it should be noted that most gait data from previous research were obtained from treadmill walking [19,20,28], which might lead to a mismatch between a participant’s visual perception and their movement pattern. For patients with gait abnormalities, there is the risk that treadmill walking restricts participants to walking in a straight line, without speed changes [29]. Therefore, in clinical gait analysis, over-ground walking is required. Limited studies have assessed the feasibility of using Kinect for gait analysis of clinical populations [27,30] and current studies mainly focus on gait analysis for healthy adults. The capabilities of Kinect to investigate abnormal gait has not been completely investigated [17] and it is unknown whether the Kinect is a feasible clinical assessment tool for children with CP. Generally, gait analysis measurement errors of 2° or less is widely accepted in clinical practice, errors between 2° to 5° are considered reasonable, and errors exceeding 5° could lead to a misleading interpretation [31]. This criterion requires that gait analysis provide subtle kinematic information, however, the poor validity proved in prior studies [17,28] indicates that the Kinect is unable to be a substitute of the current marker-based motion capture system. Nevertheless, as a portable low-cost device, Kinect might have potential applications on gait pattern prescreening, assisting observational analysis, and investigating the gait pattern progression at clinic or home-based situations. 

Therefore, the primary aim of this research is to further investigate the feasibility of using the Kinect V2 for clinical gait analysis, in children with CP. First, we aim to evaluate the concurrent validity and inter-day reliability of the Kinect, in calculating joint kinematics of over-ground gait, for children with CP, by comparing with the standard 3D gait analysis. Second, we aim to determine if calibration algorithms can improve measurement accuracy of the Kinect. We hypothesize that (1) the Kinect could provide valid and reliable joint kinematic variables, especially for the sagittal plane, and (2) calibration algorithms would improve the accuracy of Kinect-based joint kinematics. 

## 2. Materials and Methods

### 2.1. Participants

This study was approved by the local institutional review board and the University of Auckland Human Participants Ethics Committee. Children were recruited from Fujian Rehabilitation Hospital in the period of April 2018–May 2018. Inclusion criteria required the children to have a diagnosis of CP, Gross Motor Function Classification System (GMFCS) [32], Levels I to II, age 5–12 years, and with the cognitive ability to understand instructions and walking ability, to complete the gait analysis. Children were excluded if they have had significant illness, injury, or surgery within the previous six months, which might have impacted their usual activity levels in the community, or if it was not possible to complete a 3DGA. Written parental consent was obtained and written assent was given by the guardians.

Ten children were recruited for this study. The baseline characteristics are shown in Table 1.

### 2.2. Experimental Setups

Three-dimensional gait analysis data was acquired using an eight-camera Motion Analysis Eagle-4 motion capture system (Motion Analysis Corporation, Santa Rosa, CA, USA), at a sampling rate of 100 Hz. For every participant, reflective markers were placed on the sacrum, as well as the left and right anterior superior iliac spine (ASIS), thigh (cluster markers), lateral and medial condyles of the knee, the shank (cluster markers), lateral and medial malleoli of the ankle, each calcaneus, and the second metatarsal head of both feet, according to a modified Cleveland marker set [33,34]. The clusters were tied on to the participant’s lower limbs, with an elastic bandage. Marker positions were used to establish the coordinate systems for the pelvis, thigh, shank, and foot segments, and calculate the hip, knee, and ankle joint angles. 

The Microsoft Kinect v2 sensor (Microsoft Cop., Redmond, WA, USA) was placed on a tripod, at a height of 0.8 m and a distance of 5 m from the starting line. The Kinect was placed in front of the participant so that the frontal view was obtained. A previous study has suggested that the gait track should be ranged from 1.5 m to 3.5 m from the Kinect, to ensure that a minimum of one full gait cycle is captured [30]. To ensure that the steady state of gait would be recorded, the Kinect sensor was placed at a further distance than suggested. The Kinect recorder was triggered simultaneously with Motion Analysis, to collect the spatial locations of anatomical landmarks, at a fluctuating sampling frequency of around 30 Hz. The 25 anatomical landmarks, including the spine base, left/right hip, left/right knee, and left/right ankle representing the pelvis, hip, knee and ankle centers, respectively, and the left/right foot represent the toes, were recorded.

The test protocol consisted of two sessions—the first session intended to assess the validity of the Kinect and the second session intended to test the reliability. The first session was undertaken in the gait lab. After sufficient time for adaptation of the markers and walkway, participants were familiar with the testing site. They performed a static calibration trial for several seconds at their natural standing posture with their feet apart. The medial/lateral epicondyle and malleolus markers were then removed and the participants were instructed to start from the beginning of the walkway and walk barefoot towards the Kinect sensor. Each participant underwent several gait trials in the lab, at their self-preferred speeds and normal gait patterns. Three successful trials were acquired for each participant, with the criteria of a successful trial being that all reflective markers and anatomical landmarks could be seen in both motion capture interfaces. To test the reliability of the Kinect, all participants were asked to attend another Kinect gait test session in the lab, the day after. The experiment protocol for the second session was identical to the first.

### 2.3. Data Analysis

Marker position data collected by Motion Analysis were filtered through a fourth-order Butterworth low-pass digital filter, with a cut-off frequency of 6 Hz. Left/right ASIS and the sacrum determined the pelvis coordinate system. The pelvis origin was expressed as a percentage of the distance between the anterior superior iliac spines [35]. The definitions of the pelvis, thigh, shank, and foot segment coordinate systems are given in Table 2. Hip joint angles were defined as the Euler angles of the thigh coordinate system, relative to the pelvis coordinate system, rotated in a sequence of flexion/extension, adduction/abduction, and internal/external [36]. In the same way, the knee and ankle joint angles were defined as the Euler angles of the shank coordinate system, relative to the thigh coordinate system, and the foot coordinate system, relative to the shank coordinate system, rotated in the same sequence. All data reduction processes were conducted via Visual 3D Version 6 (C-Motion Inc., Germantown, MA, USA).

The anatomical landmark trajectories collected by the Kinect V2 sensor was filtered through a fourth-order Butterworth low-pass filter, with a cut-off frequency of 6 Hz. The pelvis and thigh coordinate systems were defined as conforming to the ISB recommendations [37]. The pelvis coordinate system was defined by the spine base, spine mid, hip left, and hip right markers. The left and right thigh coordinate systems were created using the corresponding knee, hip, and ankle markers. Details of the segment coordinate systems are given in Table 2. The hip joint angles were defined the same way as mentioned above. The knee and ankle angles were computed as vector angles described in Table 2. All data were processed via Matlab R2017b (MathWorks Inc., Natick, MA, USA).

To compare the differences between these two motion capture systems across the time domain, joint angles for the right limb of each gait cycle were normalized to 101 time steps. The gait cycle was represented as 0%–100%, with 0% being the initial contact of the right foot to the ground and 100% being the initial contact for the next cycle. Finally, the average joint angles of the three trials for every subject were extracted. For each degree of freedom, the maximum/minimum angle, the angle at initial contact, and the range of motion (ROM) were selected, to compare the correlation between the two systems. 

The linear regression calibration method was conducted, according to the study by Xu et al. [12]. One additional subject performed the same overground gait analysis, in which the joint angles measured by two motion capture systems were applied as an individual dataset. The linear regression equations were computed in SPSS version 25 (IBM, Armonk, NY, USA), to calibrate the joint angles. 

A standard two-layer long short-term memory (LSTM) based recurrent neural network was also implemented [38]. The network consists of 100 memory cells in each LSTM layer. The network was trained with a full batch, for each iteration, and the learning rate was set to 0.006. The gait data from the Kinect v2-based system and the 3DGA system, were constructed as three-dimensional matrices (1).

(1) $$G_{\left(n\times 101\times 5\right)}^{i}=[H_{Flex/Ext}^{i};H_{Abd/Add}^{i};H_{IntR/ExtR}^{i};K_{Flex/Ext}^{i};A_{PlantarFlex/DorsiFlex}^{i}]\;i=1\;or\;2$$

In Equation (1), gait data obtained through the Kinect based system ($G^{1}$) and the 3DGA ($G^{2}$), consisted of the hip flexion/extension angles *(*$H_{Flex/Ext}^{i}$), hip abduction/adduction angles *(*$H_{Abd/Add}^{i}$), hip internal/external rotation angles ($H_{IntR/ExtR}^{i}$), knee flexion/extension angles ($K_{Flex/Ext}^{i}$), and the ankle plantar flexion/dorsiflexion angles ($A_{PlantarFlex/DorsiFlex}^{i}$).The angles of each degree of freedom are a n×101 matrix, in which n is the experimental trials and 101 is the number of time steps.

### 2.4. Statistics

In order to evaluate the similarity and repeatability of the joint angle trajectories over a gait cycle, the coefficient of multiple correlation (CMC) was computed for each subject, following Kadaba’s approach [39]. The CMC values could be explained as—excellent similarity (0.95–1); very good similarity (0.85–0.94); good similarity (0.75–0.84); moderate similarity (0.6–0.74), and poor similarity (0–0.59) [40]. The normality of all joint angle parameters was initially tested by the Shapiro–Wilks test (*p* > 0.05). Out of the parameters which were normally distributed, the Pearson’s correlation coefficient (r) was used to assess the linear strength of association between the two motion capture methods. For the remaining non-normally distributed parameters, Spearman’s rho was used instead. The root mean square error (RMSE) was calculated to compare the differences between the two devices over a gait cycle. A Bland-Altman analysis with 95% limits of agreement (LoA) was performed to assess the agreement between two motion capture systems [41].

To assess the agreement in measurement between the two different testing sessions, the intra-class correlation coefficient (ICC) was used. The ICC was estimated, and their 95% confident intervals were calculated, using the SPSS statistical package version 25 (SPSS Inc, Chicago, IL, USA), based on a single rating and a mean rating (*k* = 3), absolute- agreement, and the 2-way random-effects model [42,43]. Point estimates of the ICC, the, *r* values were interpreted as—excellent (0.75–1), modest (0.4–0.74), or poor (0–0.39) [44]. To estimate the precision of measurement, the standard error of measurement (SEM) was calculated as:

(2) $$\mathrm{SEM}=\mathrm{SD}\times \sqrt{1-r}$$

where SD is the standard deviation of measurements determined from the ANOVA and *r* is the ICC [45,46]. Along with this, the CMC values were used to compare the angular similarity for inter-day test results. 

## 3. Results

### 3.1. Joint Kinematic Validity

**Figure 1:**
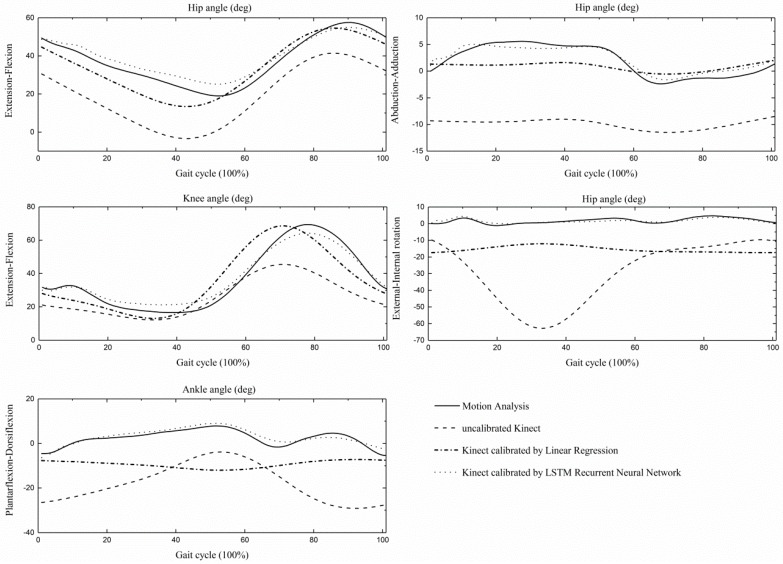
The average hip, knee, and ankle angles during a gait cycle across all gait trials.

For the integrated joint angle trajectories, ensemble curve analyses for the hip, knee, and ankle angles, during a gait cycle is shown in Figure 1 and given in Table 3. The Bland–Altman plots for every kinematic parameter are presented in Figure S1 (Supplementary Materials); mean difference, LoA, Upper and Lower LoA are given in Tables S1–S3 (Supplementary Materials). From our results, we can see that only the CMC values for the knee flexion/extension angle reached moderate similarity (0.70 ± 0.12), whereas it was poor for both the hip and ankle angles. After both calibration methods, the CMC values increased for the knee, hip sagittal, and frontal angles. The similarity at the hip and knee flexion/extension angles increased to 0.81(0.10)/0.75(0.22) and 0.85(0.07)/0.87(0.12) respectively, which implied good and very good similarity. The hip rotation and ankle flexion trajectories had poor similarity with its reference, after the two calibration methods; however, the LSTM method could slightly improve the ankle flexion CMC value (0.43 ± 0.38). Concurrently, all RMSE values decreased, after both calibration methods, in which the hip internal/external rotation reduced the most. Similarly, as presented in Tables S1–S3, the mean of difference in every degree of freedom (DOF) decreased after calibration, when comparing the two calibration methods, the results applying the LSTM method had the smallest mean difference. The smallest difference between the two devices existed in the hip frontal angles, under both uncalibrated and calibrated conditions. Comparatively, the largest errors existed in the hip internal/external rotation angles, under calibrated conditions. 

The joint kinematic variables, mean (±SD) values, and correlation relationships between the two systems for each parameter are presented in Table 4. For kinematics variables that were uncalibrated and calibrated by linear regression method, the overall correlation was poor to modest (−0.17, 0.72) between the two systems, while the minimum ankle flexion angle (0.77) and ankle flexion angle at initial contact (0.75) yielded an excellent correlation. Moreover, there were several parameters, including the hip sagittal (0.55) and transverse ROM (0.58), the maximum hip frontal angle (0.72), the initial knee flexion/extension angle (0.41), and knee ROM (0.43), which showed modest agreement with the 3D motion capture system. In terms of the LSTM calibration method, poor relative agreement (−0.86, 0.37) for all the kinematics variables was observed, except for the knee ROM (0.59) and the ankle ROM (0.49). 

### 3.2. Joint Kinematic Reliability

**Figure 2:**
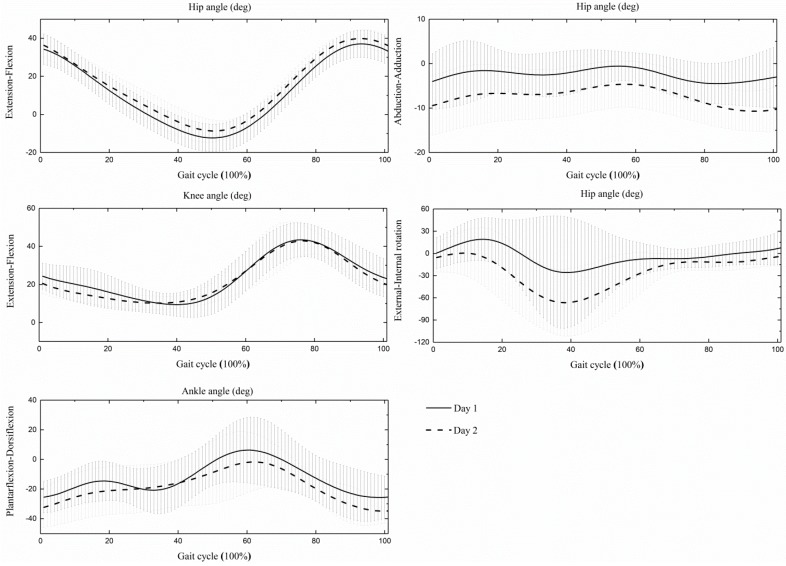
The average (±SD) hip, knee, and ankle angles during a gait cycle, across all walking trails in day 1 and day 2.

The integrated joint angle trajectories comparison between two days are depicted in Figure 2 and summarized in Table 5. The between-day hip flexion/extension angle trajectories reached excellent similarity (0.97 ± 0.05) and the knee flexion/extension angle trajectories indicated very good similarity (0.88 ± 0.12), while the other joint angle trajectories showed a poor similarity. The largest RMSE was at the hip internal/external angle (43.4° ± 36.4°), and the smallest measurement error existed at the hip sagittal angle (5.3° ± 2.6°).

In terms of joint kinematic parameters, the mean (±SD) values and the results for inter-day reliability are listed in Table 6. Results showed that reliability for most variables was modest to excellent (−0.23, 0.93); among them, the hip sagittal ROM and the initial contact joint angle reached 0.87 and 0.76, respectively. Generally, almost all hip sagittal variables showed a better reliability than that of the other angles.

## 4. Discussion

The validity of kinematics of children with CP determined from the Kinect is relatively poor, compared to that obtained from the traditional marker-based 3DGA system. While after applying the linear regression and LSTM calibration methods, the measurement errors of every ROM decreased, the hip and knee sagittal angle trajectories, especially closely resembled the reference marker-based motion capture system. Although calibration algorithms improved the performance of Kinect, it should also be noted that the Kinect could not provide subtle kinematic information. It can be seen from Figure 1, that the hip joint angles measured by the Kinect underestimated the hip flexion angle and overestimated the hip extension angle, which was consistent with the findings from previous Kinect-based treadmill and over-ground gait validation studies [18,19]. There are several possible reasons for the differences between the two systems. First, for marker-based motion capture, the hip joint center was located at a fixed distance between the anterior superior iliac spines [35], and the pelvis orientation was defined by the anterior and posterior iliac spines. Therefore, the pelvis orientation was very sensitive to the relative positions between these anatomical markers, which were determined by the characteristics of the individual’s pelvic anatomical structure. For children with CP, the imbalance between the hip flexor and the extensor, balance problems and distal deformity might have led to abnormal pelvic tilt angles, which represented the pelvic position in the sagittal plane [47]. Previous studies found that the Kinect-observed pelvis was wider and had a low correlation with what was captured by a 3D camera method, in vertical and anterior-posterior axis, which substantiated the fact of the underestimated inter-subject pelvic tilt variability [8,18,19,48,49]. The Kinect had a limited ability to determine a non-joint center related orthogonal axis to the longitudinal axis [8]. In this study, the dissimilarity of the Kinect-detected pelvic landmarks and its counterpart, predicted with the marker-based motion capture system, possibly led to the measurement differences between the two systems. A recent study manually marked the same anatomical markers as that in the 3D motion capture system, based on visual judgments of the depth images, and as an outcome, showed good to excellent validity results for the single leg squat task [14]. It was more expedient and clinically viable for using skeletal landmarks directly. However, these promising results indicate that future studies could also apply depth image data as additional information to help achieve a better result. 

The second possible explanation for the measurement differences might be due to the different definitions of the femur coordinate system. It is known that at least three non-colinear markers are required to determine a rigid body segment [50]. Unlike the marker-based motion capture system, we had to make a compromise when defining segments with limited skeletal landmarks. In this study, the ankle landmark was invoked to define the femur coordinate system, when only two landmarks (hip and knee landmark) were available. Similar adjustments were also made when defining the shank and foot segment, and the joint angles were defined as the angle between two vectors, rather than an Euler angle. Although the correlation between the two systems was poor to modest, the calibrated hip and knee sagittal angular wave showed good similarity with the 3D motion capture method. Since most observational gait classification studies only used the sagittal data [51], the Kinect has the potential to assist in observational gait analysis. In terms of the frontal and transverse variables, which were also significant for clinical treatment and intervention decision making, the Kinect was incompetent in providing rational results, even with the calibration process. 

According to the findings of Mentiplay et al., placing the Kinect in front of subjects could ensure spatiotemporal accuracy, whilst obtaining bilateral lower limb kinematics in a clinically feasible single trial. Their pilot experiences revealed that the placement of the Kinect was not a sensitive factor in influencing the results [18]. Another similar study reported that the optimal position for sagittal kinematic tracking for the Kinect to avoid treadmill obstruction in the walking direction, was at a 45° angle [28]. Since our study addressed the over-ground gait analysis, treadmills and other obstacles were non-existent, and so the Kinect was placed, aligned with the patients’ walking direction. Future studies can compare the different Kinect placement protocols to meet various clinical requirements and provide recommendations for clinical application. Furthermore, whether or not a participants clothing affects the validation of results, still remains ambiguous. Further work could continue to improve the test protocol for the clinical application of the Kinect.

The poor validation results on the ankle in this study were a widespread phenomenon among several studies [18,20,52]. Otte et al. found that the foot landmark showed the poorest tracking accuracy in three axes, when compared with other skeletal landmarks [48]. Unlike other body segments, the foot is located in the anterior–posterior direction of the body. Being accompanied with ground light reflection, tracking errors might occur [53]. According to Dubois et al., a feasible solution for this is to extract gait variables from the geometric centers of depth images, which could partly avoid foot tracking visual occlusions [54,55]. Along with this, the LSTM calibration method provided in this study shows the possibility of improving ankle kinematics. Further progressive updates in foot tracking accuracy are needed, if the Kinect is utilized in a clinic or home-based condition.

The results show that the hip and knee sagittal angles were more accurate. The efficacy of the two calibration methods was similar at the hip frontal/sagittal plane and knee sagittal plane. Additionally, the LSTM calibration method had the advantage of improving the ankle tracking accuracy. Both of the above-mentioned calibration methods could be used to improve the lower extremity kinematics. When compared with the LSTM-based calibration method, the linear regression method has a lower experimental and computational expenditure, but an equivalent calibration efficacy for hip and knee angles. Meanwhile, the neural network-based calibration method shows the potential to address poor foot tracking problems. The diversity of abnormal gait deviations observed in children with CP require the incorporation of large, prospective, population-based cohorts, and classified categories [51], from which the LSTM-based calibration procedure could benefit. A prior study showed the overestimation in shoulder frontal kinematics, with the Kinect and the linear regression calibration method, which helped to reduce the measurement errors [12]. In our study, the linear regression method could be used to minimize the underestimation in lower limb kinematics, in a cost-effective way.

We found that the inter-day hip sagittal angle trajectories had excellent similarity, and modest to excellent reliability, while the inter-day knee sagittal angular curve showed a very good similarity and a modest to poor reliability. Other kinematic parameters exhibited a poor to modest reliability in this study. There was only one study reporting the inter-day gait kinematic reliability of the Kinect, and they investigated that the reliability was modest to poor for most kinematic variables, except for peak knee flexion angle, during swing, which showed a negative correlation coefficient [18]. However, our results proved that the reliability of the peak knee flexion angle was modest. The different clinical population is an obvious cause of the difference in interpretation between this study and the previous one. Adult gait data were generally considered to be less variable than children’s [56], while children with CP was more variable than their healthy peers [57]. Although a poor foot tracking accuracy was observed in the validity assessment, it is notable that the ankle range of motion reliability was modest, which is consistent with the prior study [18]. To some extent, it proved that the tracking error was not a random phenomenon. The reliability of ankle kinematics has the possibility to be improved with the development of foot tracking techniques. We found that lower CMC existed at the frontal and transverse planes, and higher CMC values existed at the sagittal plane. The same phenomenon also happened in the optoelectronic 3D gait analysis [31,34]. The markerless tracking strategy gives the Kinect advantages in avoiding the between-assessor variance. With a similar level of reliability, the Kinect can be an option for the clinic or home-based observation, which could help track gait pattern progression, with or without treatment.

## 5. Limitations

Despite the promising results, this study had some limitations. Gait analysis was obtained from ten children with CP. This was a relative smaller number than that of uninvolved outpatients in the local hospital. The primary reason of this poor recruitment was that a large proportion of the children were excluded, because they did not meet the inclusion criteria. Second, more patients with various CP subtypes, GMFCS levels, and abnormal gait patterns should be recruited to confirm the obtained results in future studies. In addition, a larger population could also facilitate a more in-depth investigation of using the LTSM recurrent neural network, to improve the Kinect-based kinematics. 

## 6. Conclusions

As the first study to investigate abnormal gait in children with CP using the Kinect, this piece of work reveals that although the overall poor validity of the Kinect has been investigated, the Kinect-based gait analysis system has the potential to improve its ability of measuring lower limb kinematics. Future attempts could be made to reduce measurement errors across the following aspects—(1) developing enhanced calibration methods and involving more participants with different gait patterns to constitute a specific database; (2) establishing a standard experimental protocol for specific clinical groups; (3) adding informative depth image data to improve kinematic computation. Reliable hip and knee sagittal angles could provide a portable option for clinics or home-based investigations surrounding ongoing changes in gait. Moreover, the Kinect generally demonstrates a feasible reliability and comparable validity in some spatiotemporal parameters [17,18,30]. These promising results support the Kinect’s clinical capacity in becoming a potential gait analysis tool, with more refinement of the system.

## Figures and Tables

**Table 1:** Baseline characteristics of the ten participants, values are means (SD).

Characteristics	Experimental Group (n = 10)
Age, y	6.4 (2.2)
Weight, kg	23.2 (7.4)
Height, cm	116.7 (11.0)
Gender, M/F	3/7
GMFCS level, n	
I	3
II	7
Type of CP, n	
Hemiplegia	1
Diplegia	5
Quadriplegia	2
Dyskinetic	2

**Table 2:** Definitions of segment coordinate systems.

Segment	Kinect	Motion Analysis
Right pelvis	Origin: spine base marker; *y*-axis: unit vector from the spine base to spine mid; *x*-axis: cross-product of *y*-axis and the unit vector from hip left to hip right; *z*-axis: cross product of *x* and *y*-axis.	Origin: mid-point between the mid-anterior superior iliac spines and sacrum; *x*-axis: unit vector from the origin to the right anterior superior iliac spine; *z*-axis: unit vector perpendicular to the plane formed by the anterior superior iliac spines and sacrum); *y*-axis: cross product of *z* and *x*-axis.
Right thigh	Origin: knee right marker; *y*-axis: unit vector from knee right to hip right; *z*-axis: cross-product of *y*-axis and the vector from ankle right to knee right; *x*-axis: cross-product of *y* and *z*-axis.	Origin: mid-point between lateral and medial knee markers; *z*-axis: unit vector from the origin to right pelvis origin; *y*-axis: unit vector perpendicular to the plane which formed by right hip origin, lateral and medial knee markers; *x*-axis: cross product of *y* and *z*-axis.
Right shank	The knee angle calculated by Kinect equaled the supplementary angle of the angle between vector pointing from hip right to knee right and vector pointing from knee right to ankle right.	Origin: mid-point between lateral and medial ankle markers; *z*-axis: unit vector from origin to right thigh origin; *y*-axis: unit vector perpendicular to the plane formed by right thigh origin, right lateral and medial ankle markers; *x*-axis: cross product of *y* and *z*-axis.
Right foot	The ankle angle calculated by Kinect was the angle between the vector pointing from ankle right to knee right and the vector pointing from ankle right to foot right subtract 90°.	Origin: the 2nd metatarsal joint; *y*-axis: unit vector from the origin to right shank origin; *x*-axis: unit vector perpendicular to the plane formed by heel, origin and right shank origin; *y*-axis: cross product of *z* and *x*-axis.

**Table 3:** Root mean square error (RMSE), coefficients of multiple correlation (CMC) (±SD) between the joint angle trajectories, using Kinect, Kinect calibrated by linear regression (LR) and long short-term memory (LSTM) recurrent neural network, and Motion Analysis.

Joint Angles	RMSE (degree)			CMC		
	Kinect	Calibrated by LR	Calibrated by LSTM	Kinect	Calibrated by LR	Calibrated by LSTM
Hip flexion/extension	20.7 ± 8.8	11.5 ± 4.1	11.2 ± 4.9	0.45 ± 0.36	0.81 ± 0.10	0.75 ± 0.22
Hip abduction/adduction	12.5 ± 3.4	4.7 ± 2.2	5.2 ± 1.7	<0.001	0.41 ± 0.35	0.42 ± 0.37
Hip int/external rotation	40.2 ± 22.6	18.0 ± 8.7	10.3 ± 4.6	<0.001	<0.001	<0.001
Knee flexion/extension	16.7 ± 4.2	14.1 ± 4.8	10.5 ± 5.1	0.70 ± 0.12	0.85 ± 0.07	0.87 ± 0.12
Ankle dorsi/plantarflexion	23.0 ± 5.0	13.7 ± 5.8	7.5 ± 3.0	<0.001	<0.001	0.43 ± 0.38

**Table 4:** Mean (±SD) values for each kinematic variables, Pearson (*r*)/Spearman’s correlation coefficient (*r_s_*) between the kinematic variables, calculated by Kinect, Kinect calibrated by linear regression (LR) and LSTM, and Motion Analysis.

	Joint Angles				*r*/*r_s_*	
	Kinect	Motion Analysis	Calibrated by LR	Calibrated by LSTM	Kinect/Calibrated by LR	Calibrated by LSTM
**Hip flexion/extension**						
maximum	42.4 ± 6.6	57.8 ± 7.2	55.5 ± 6.0	56.2 ± 5.4	0.02	−0.02
minimum	−4.2 ± 7.6	14.1 ± 9.3	12.6 ± 7.0	24.2 ± 5.7	0.20 *^a^*	−0.18
ROM	46.6 ± 11.4	39.7 ± 9.7	42.9 ± 10.5	32.0 ± 6.5	0.55	0.26
initial contact	30.5 ± 8.2	49.4 ± 8.3	44.6 ± 7.5	48.7 ± 1.4	0.19	−0.51
**Hip adduction/abduction**						
maximum	−5.2 ± 4.2	7.4 ± 5.7	4.9 ± 3.7	7.5 ± 2.1	0.72 *^a, b^*	0.37
minimum	−14.2 ± 4.8	−5.0 ± 4.1	−2.9 ± 4.2	−3.6 ± 2.7	0.26	−0.25 *^a^*
ROM	9.0 ± 5.4	12.3 ± 7.3	7.8 ± 4.7	11.2 ± 3.4	0.15^*a*^	−0.67
initial contact	−9.3 ± 5.8	−0.003 ± 5.0	1.3 ± 5.1	1.0 ± 0.9	0.37	−0.64 *^b^*
**Hip int/external rotation**						
maximum	1.4 ± 13.7	7.3 ± 8.7	−11.6 ± 4.4	7.7 ± 6.6	0.12	−0.23
minimum	−67.9 ± 43.7	−4.5 ± 10.4	−18.5 ± 1.4	−4.7 ± 3.5	0.12	−0.22
ROM	69.3 ± 46.3	11.9 ± 6.5	6.9 ± 4.6	12.4 ± 5.5	0.58	0.18 *^a^*
initial contact	−9.8 ± 21.2	0.1 ± 10.9	−17.4 ± 2.1	0.4 ± 1.4	−0.17	−0.86 *^c^*
**Knee flexion/extension**						
maximum	46.7 ± 7.5	71.2 ± 8.6	70.6 ± 12.5	65.9 ± 6.8	0.32	0.28
minimum	10.8 ± 5.8	15.3 ± 10.4	10.7 ± 9.7	18.1 ± 6.0	0.14 *^a^*	0.13
ROM	35.9 ± 10.3	55.9 ± 16.2	60.0 ± 17.1	47.7 ± 10.5	0.43	0.59
initial contact	21.1 ± 9.1	31.7 ± 8.1	27.9 ± 15.2	30.0 ± 1.1	0.41	−0.02
**Ankle dorsi/plantar flexion**						
maximum	1.3 ± 6.3	10.5 ± 6.9	−6.2 ± 1.2	10.2 ± 3.7	0.09	0.04
minimum	−34.7 ± 6.2	−7.8 ± 7.6	−13.0 ± 1.2	−7.8 ± 2.1	0.77 *^c^*	−0.10
ROM	36.0 ± 7.0	18.3 ± 6.0	6.8 ± 1.3	18.0 ± 4.9	0.26	0.49
initial contact	−26.6 ± 8.6	−4.6 ± 6.5	−7.7 ± 1.6	−6.4 ± 0.9	0.75 *^b^*	−0.72 *^b^*

*^a^* the Spearman’s rank correlation coefficient; *^b^ p* < 0.05; *^c^ p* < 0.01.

**Table 5:** RMSE, CMC (±SD) between the joint angle trajectories calculated by Kinect in day 1 and day 2.

Joint Angles	RMSE (degree)	CMC
Hip flexion/extension	5.3 ± 2.6	0.97 ± 0.05
Hip abduction/adduction	6.6 ± 3.6	0.30 ± 0.36
Hip internal/external rotation	43.4 ± 36.4	0.40 ± 0.43
Knee flexion/extension	7.9 ± 3.6	0.88 ± 0.12
Ankle dorsi/plantarflexion	17.5 ± 9.6	0.44 + 0.40

**Table 6:** Mean (+SD) values for each parameter testing in day 1 and day 2. Reliability (intra-class correlation coefficients (ICC*_2,k_*) with 95% confidence intervals for agreement and standard error of measurement (SEM).

	Joint Angles		ICC*_2,k_*	SEM
	Day 1	Day 2		
**Hip flexion/extension**				
maximum	37.5 ± 7.3	40.3 ± 4.3	0.77(0.16, 0.94)	2.89
minimum	13.1 ± 7.2	9 ± 5.7	0.80(0.09, 0.95)	2.94
ROM	50.6 ± 8.6	49.5 ± 6.7	0.93(0.73, 0.98)	1.83
initial contact	34.2 ± 7.9	36.3 ± 6.1	0.86(0.49, 0.97)	2.78
**Hip adduction/abduction**				
maximum	3.0 ± 5.0	2.1 ± 5.3	0.46(−0.38, 0.84)	4.16
minimum	8.3 ± 2.9	12.7 ± 4.4	0.21(−0.56, 0.74)	3.81
ROM	11.3 ± 4.0	10.6 ± 3.5	0.59(−0.79, 0.90)	2.34
initial contact	4.0 ± 6.3	9.4 ± 6.7	0.53(−0.34, 0.87)	4.75
**Hip internal/external rotation**				
maximum	35.7 ± 34.6	11.8 ± 28.4	0.37(−0.72,0.82)	26.36
minimum	50.5 ± 50.9	69.9 ± 45.5	−0.23(−4.69,0.70)	53.37
ROM	86.2 ± 56.1	81.7 ± 39.3	0.64(−0.63, 0.91)	28.37
initial contact	0.7 ± 20.9	5.4 ± 20.0	0.46(−1.26, 0.87)	14.86
**Knee flexion/extension**				
maximum	45.1 ± 9.0	45.6 ± 7.2	0.62(−0.73, 0.91)	4.87
minimum	8.3 ± 6.0	8.0 ± 2.1	0.48(−1.51, 0.88)	3.15
ROM	36.7 ± 8.74	37.6 ± 6.5	0.36(−2.2, 0.85)	6.02
initial contact	24.3 ± 6.8	20.6 ± 5.5	0.45(−0.70, 0.85)	4.69
**Ankle dorsi/plantar flexion**				
maximum	9.8 ± 21.1	3.7 ± 17.1	0.50(−1.0, 0.88)	13.42
minimum	−33.8 ± 7.4	−37.4 ± 8.1	0.05(−2.7, 0.76)	7.59
ROM	43.6 ± 22.0	41.2 ± 13.4	0.62(−0.72, 0.91)	11.03
initial contact	−25.5 ± 10.6	−32.3 ± 13.4	0.27(−1.39, 0.81)	10.49

## Supplementary Materials

The following are available online at https://www.mdpi.com/1424-8220/19/7/1660/s1, Figure S1. Bland-Altman plots with 95% limits of agreement (LoA). X axes represents the angle means of two systems and the Y axes represents the mean of differences. The red line (middle one) represents the reference line at mean, and the two dashed lines represent the upper and lower limit of agreement. From left to right, the plots are measurement differences between “Kinect vs. Motion Analysis”, “Kinect calibrated by linear regression vs. Motion Analysis” and “Kinect calibrated by LSTM vs Motion Analysis” respectively. Table S1. Results of Bland-Altman analysis of agreement between joint kinematic parameter calculated from Kinect and Motion Analysis. Mean difference, limits of agreement (LoA), Lower LoA and Upper LoA are reported. Table S2. Results of Bland-Altman analysis of agreement between joint kinematic parameter calculated from Kinect (calibrated by linear regression method) and Motion Analysis. Mean difference, limits of agreement (LoA), Lower LoA and Upper LoA are reported. Table S3. Results of Bland-Altman analysis of agreement between joint kinematic parameter calculated from Kinect (calibrated by long short-term memory recurrent neural network) and Motion Analysis. Mean difference, limits of agreement (LoA), Lower LoA and Upper LoA are reported.
