# Supplementary material for: The Validity and Reliability of a Kinect v2-Based Gait Analysis System for Children with Cerebral Palsy
Source: Sensors (Basel). 2019 Apr 7;19(7):1660. doi: 10.3390/s19071660 (PMC6479781; doi:10.3390/s19071660)
Supplement: Supplementary file 1 [file sensors-19-01660-s001.pdf]

Supplementary Material – Bland-Altman plots for joint kinematics parameters during gait.

Maximum Hip flexion/extension angle

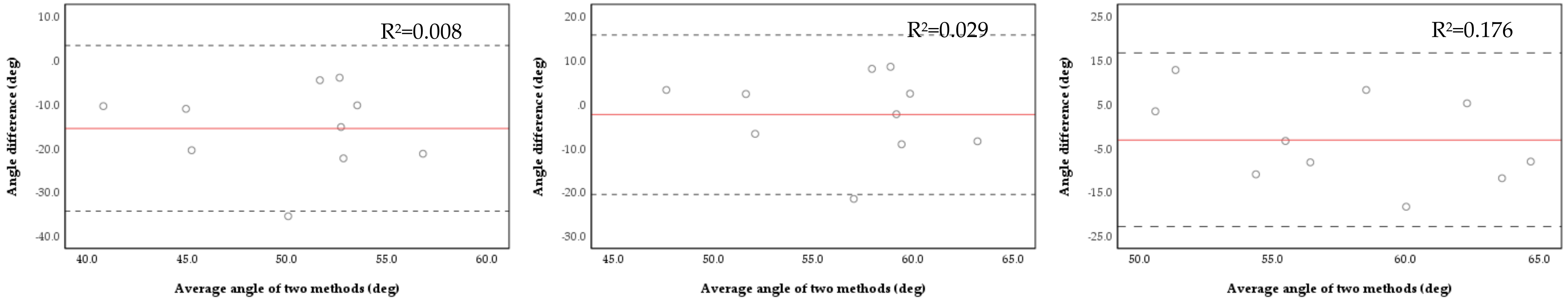

Minimum Hip flexion/extension angle

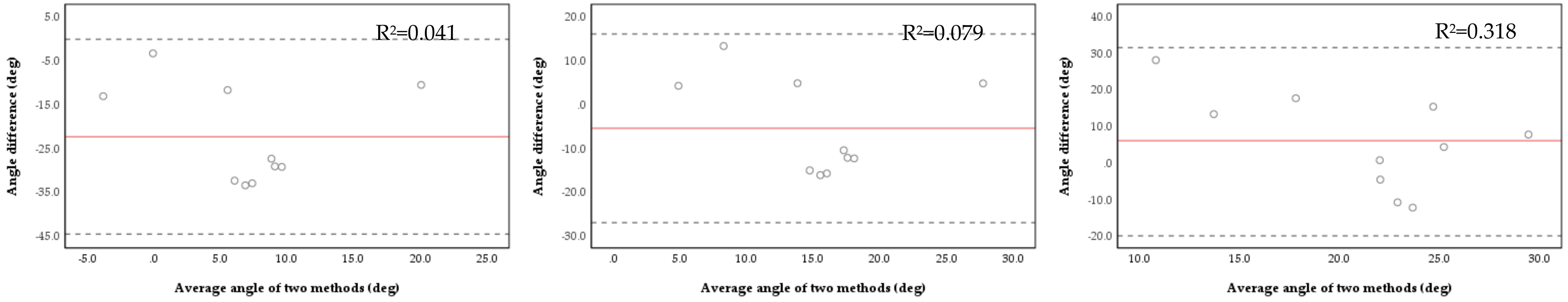

Hip flexion/extension ROM

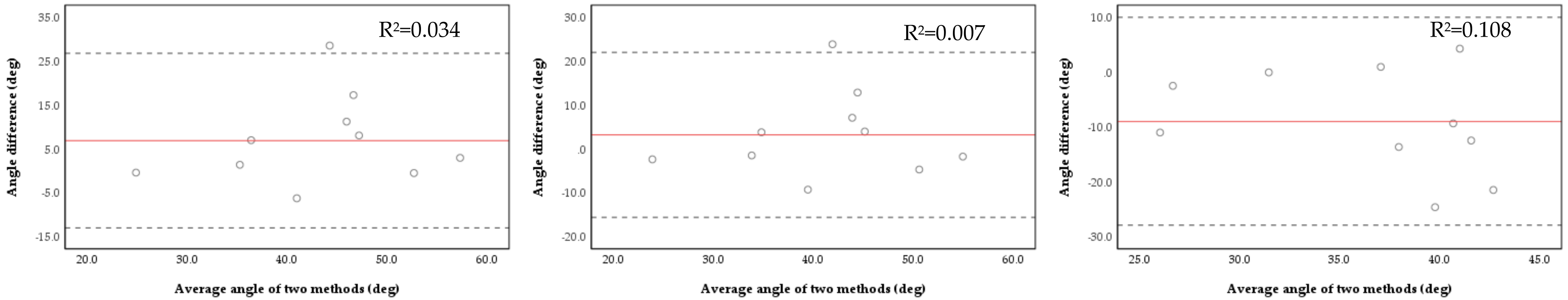

Hip flexion/extension at initial contact

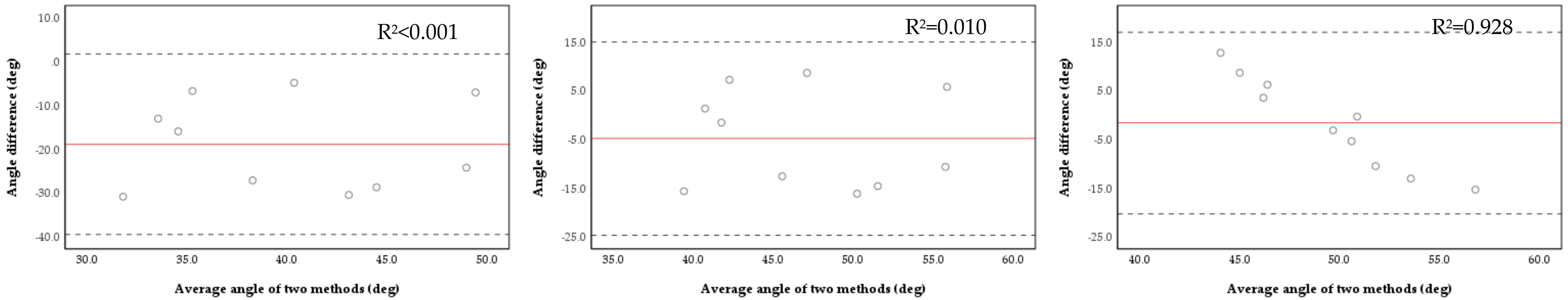

Maximum Hip adduction/abduction angle

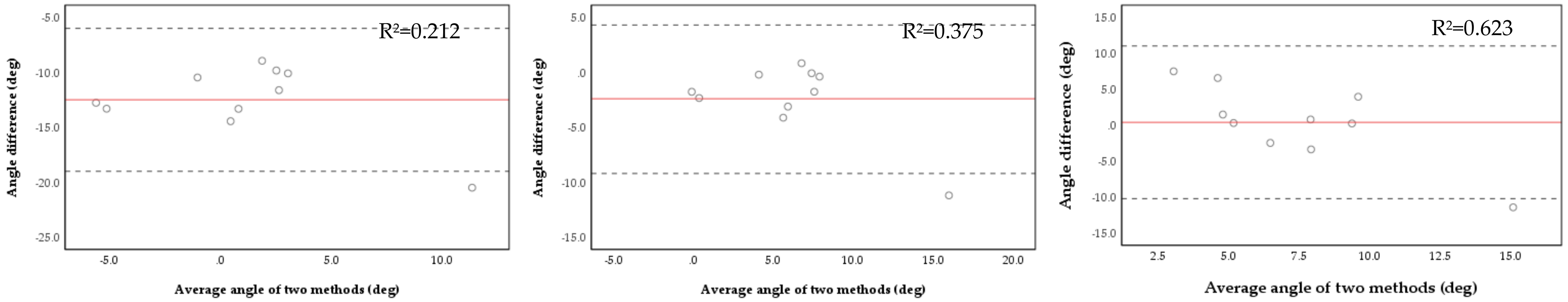

Minimum Hip adduction/abduction angle

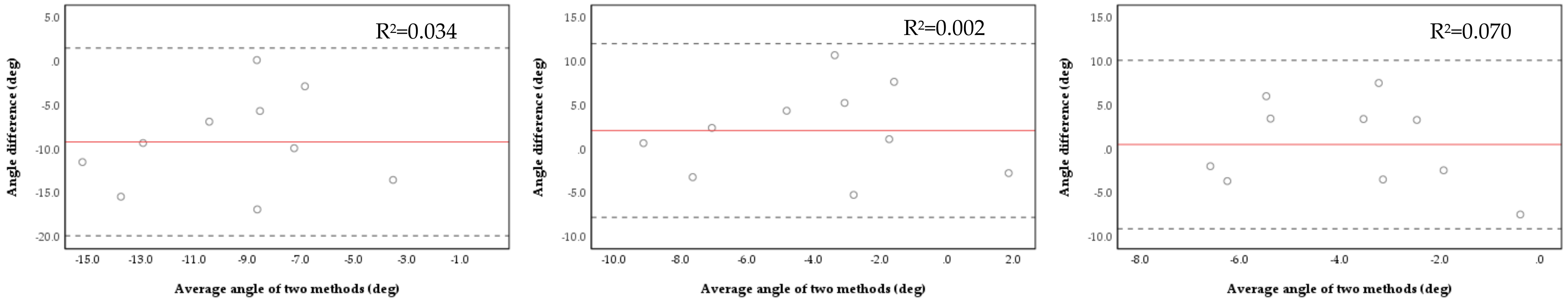

Hip adduction/abduction ROM

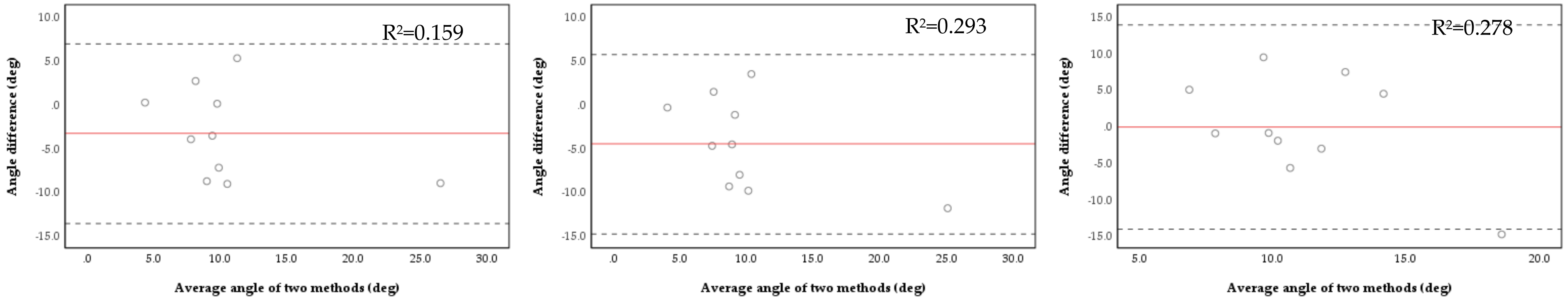

Hip adduction/abduction at initial contact

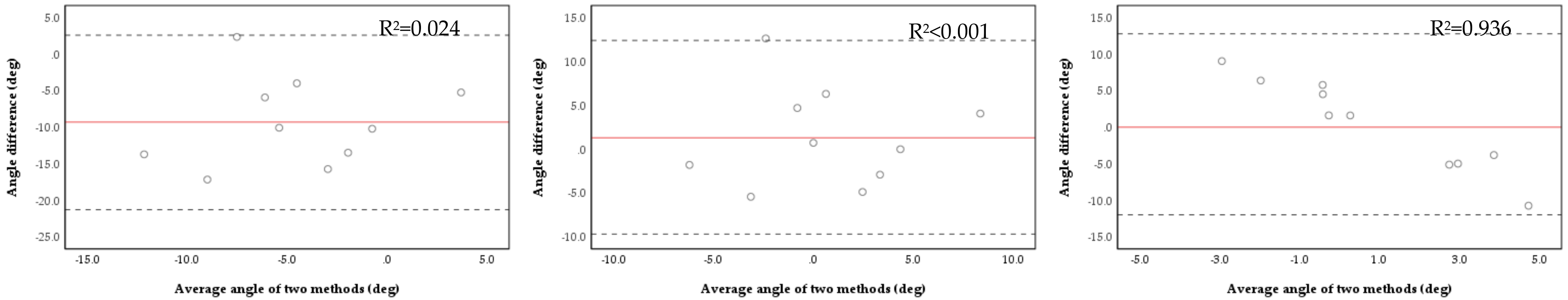

Maximum Hip internal/external rotation angle

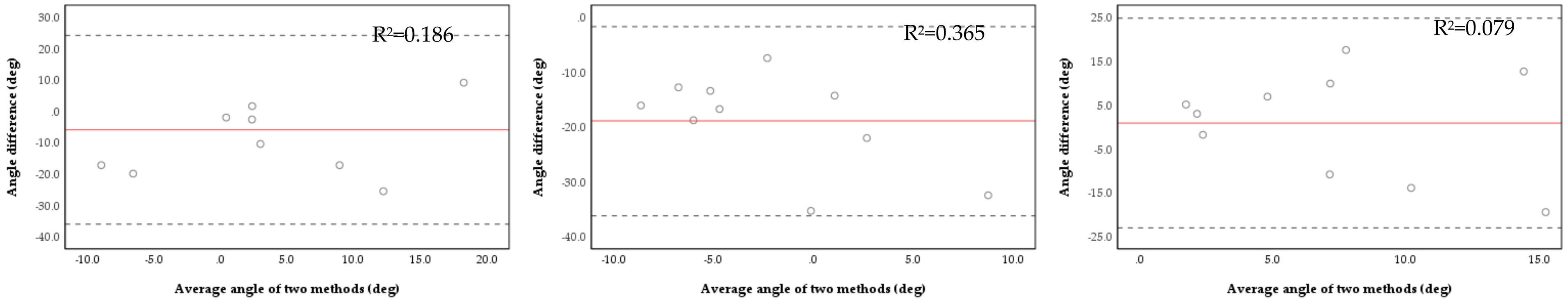

Minimum Hip internal/external rotation angle

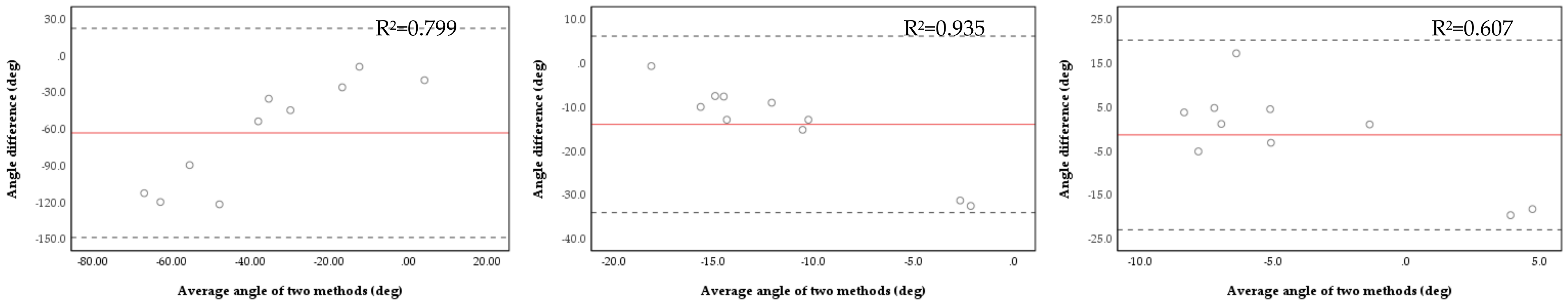

Hip internal/external rotation ROM

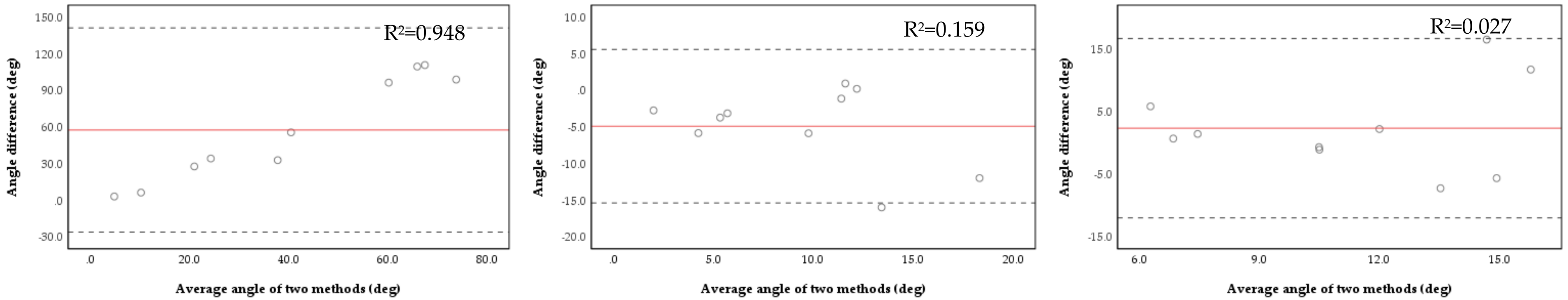

Hip internal/external rotation at initial contact

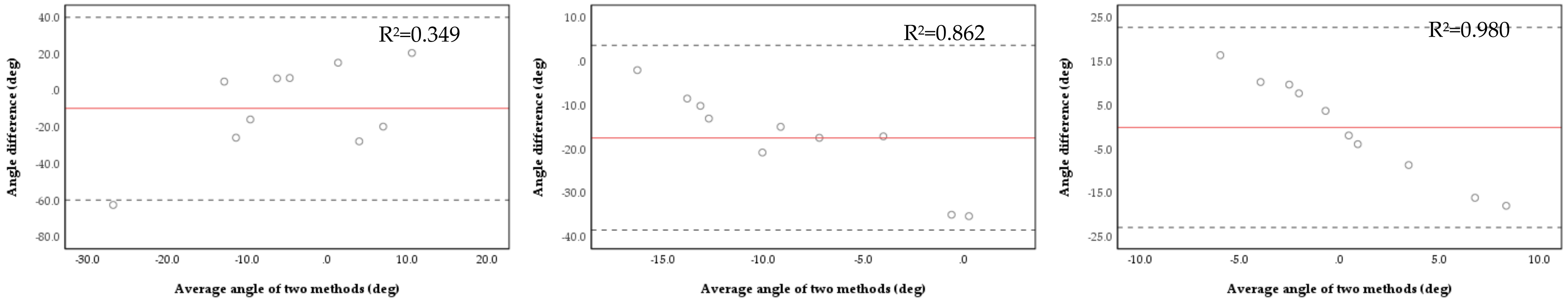

Maximum Knee flexion/extension angle

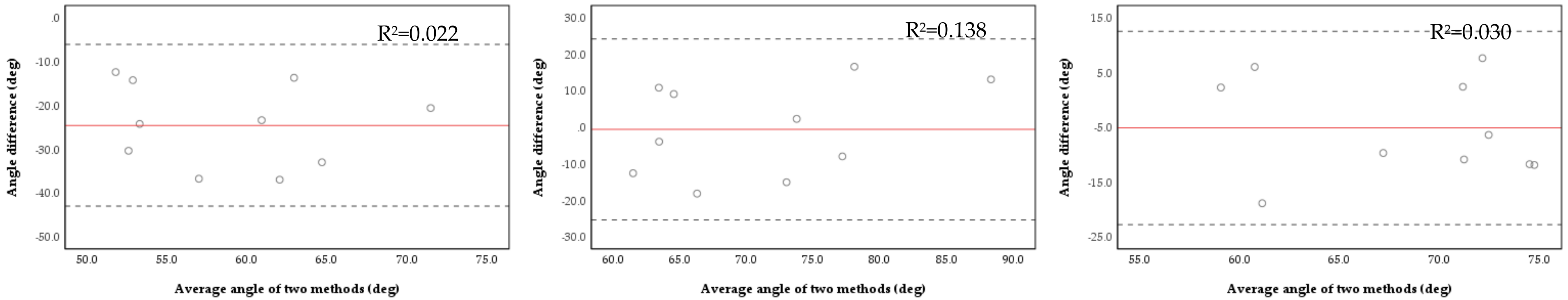

Minimum Knee flexion/extension angle

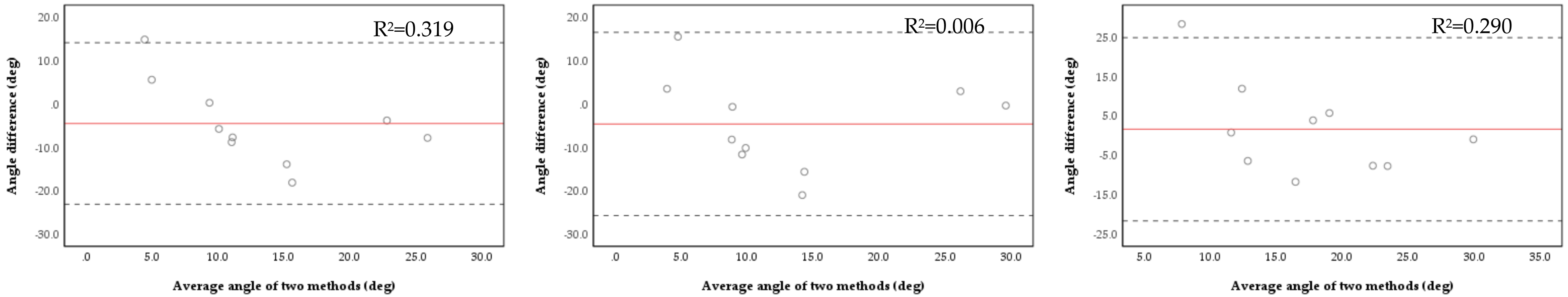

Knee flexion/extension ROM

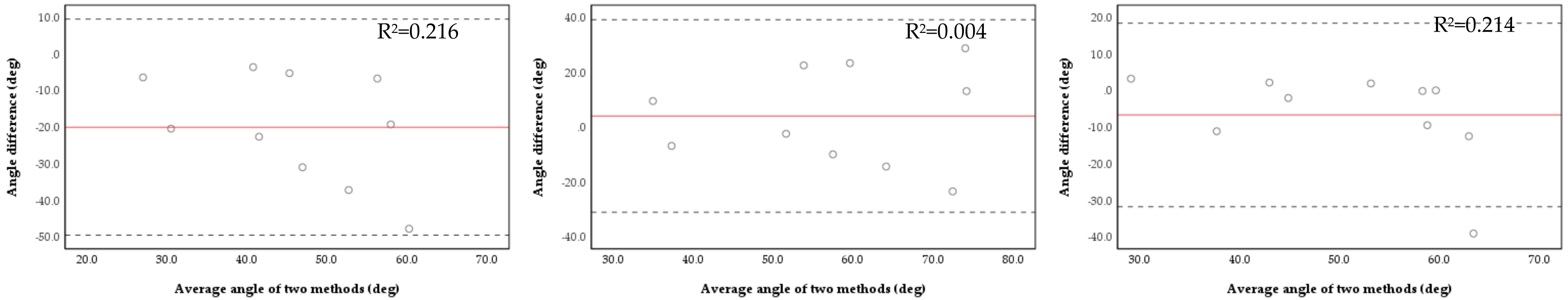

Knee flexion/extension angle at initial contact

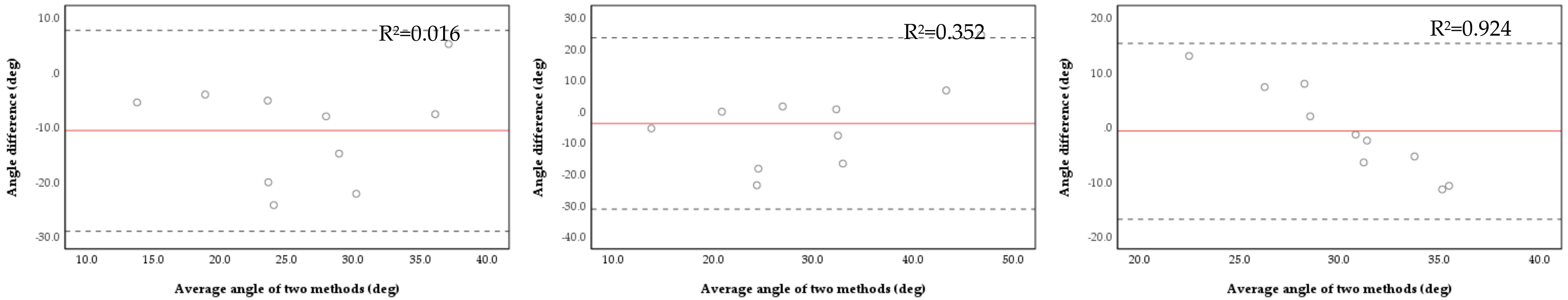

Maximum Ankle dorsiflexion/plantarflexion angle

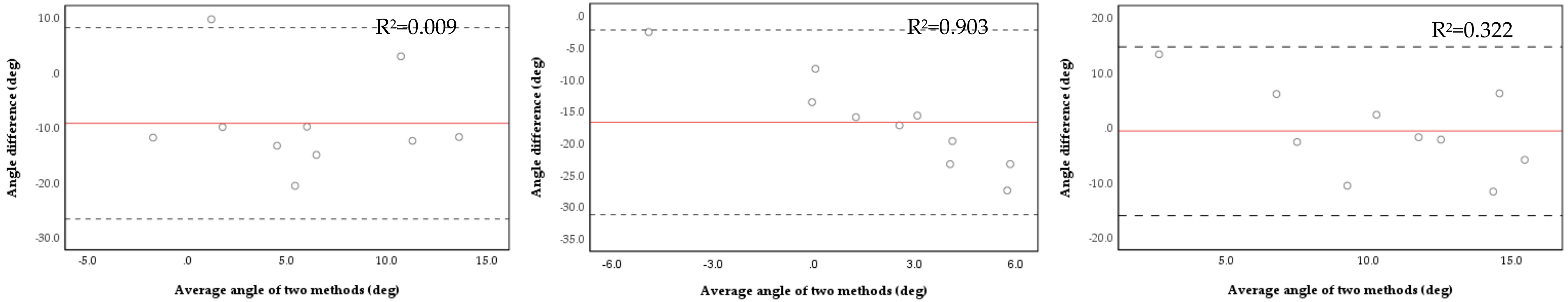

Minimum Ankle dorsiflexion/plantarflexion angle

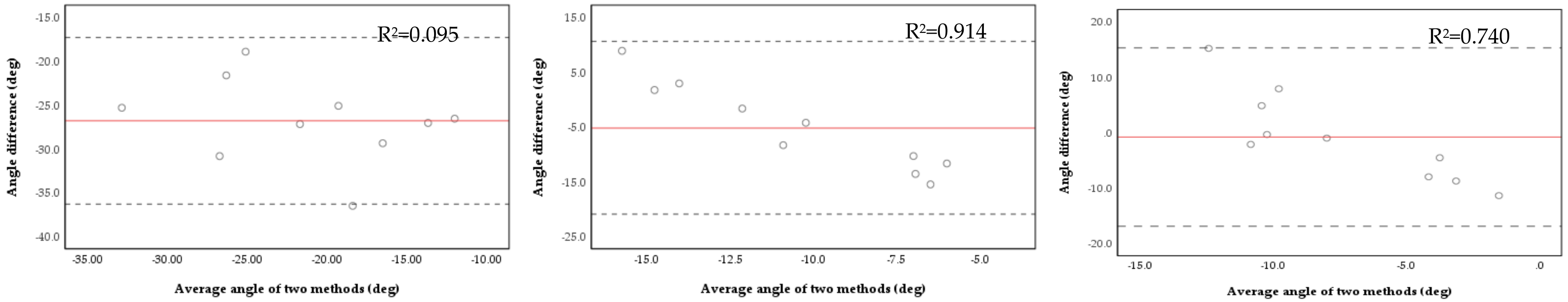

Ankle dorsiflexion/plantarflexion ROM

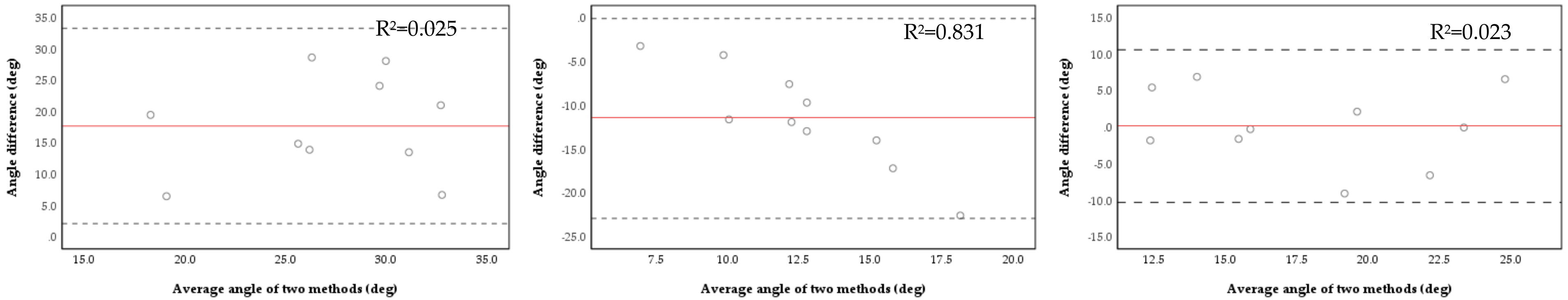

Ankle dorsiflexion/plantarflexion at initial contact

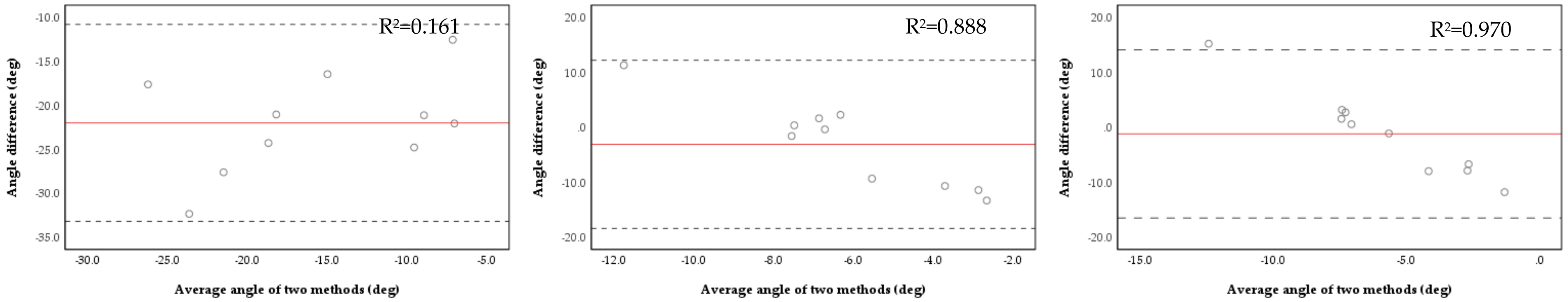

Figure 1S. Bland-Altman plots with 95% limits of agreement (LoA). X axes represents the angle means of two systems and the Y axes represents the mean of differences. The red line (middle one) represents the reference line at mean, and the two dashed lines represent the upper and lower limit of agreement. From left to right, the plots are measurement differences between "Kinect vs. Motion Analysis", "Kinect calibrated by linear regression vs. Motion Analysis" and "Kinect calibrated by LSTM vs Motion Analysis" respectively.

Table S1. Results of Bland-Altman analysis of agreement between joint kinematic parameter calculated from Kinect and Motion Analysis. Mean difference, limits of agreement (LoA), Lower LoA and Upper LoA are reported.

Kinematic parameter	Mean difference	LoA	Lower LoA	Upper LoA
**Hip flexion/extension**				
maximum	-15.5	18.8	-34.3	3.4
minimum	-22.4	22.2	-44.6	-0.2
ROM	6.9	19.9	-12.9	26.8
initial contact	-18.9	20.6	-39.5	1.7
**Hip adduction/abduction**				
maximum	-12.5	6.5	-19.0	-6.0
minimum	-9.2	10.7	-19.9	1.5
ROM	-3.3	10.2	-13.6	6.9
initial contact	-9.3	11.9	-21.3	2.6
**Hip int/external rotation**				
maximum	-5.9	30.1	-36.0	24.2
minimum	-63.4	85.7	-149.1	22.3
ROM	57.5	83.8	-26.3	141.3
initial contact	-9.8	49.8	-59.6	40.0
**Knee flexion/extension**				
maximum	-24.6	18.4	-43.0	-6.1
minimum	-4.5	18.6	-23.1	14.1
ROM	-20.1	29.5	-49.6	9.5
initial contact	-10.6	18.4	-29.0	7.7
**Ankle dorsi/plantar flexion**				
maximum	-9.2	17.4	-26.6	8.2
minimum	-26.8	9.5	-36.3	-17.3
ROM	17.7	15.6	2.1	33.3
initial contact	-22.0	11.2	-33.2	-10.8

Table S2. Results of Bland-Altman analysis of agreement between joint kinematic parameter calculated from Kinect (calibrated by linear regression method) and Motion Analysis. Mean difference, limits of agreement (LoA), Lower LoA and Upper LoA are reported.

Kinematic parameter	Mean difference	LoA	Lower LoA	Upper LoA
**Hip flexion/extension**				
maximum	-2.3	9.3	-20.5	15.8
minimum	-5.5	11.0	-27.0	16.0
ROM	3.2	9.6	-15.6	22.0
initial contact	-4.8	10.1	-24.7	15.0
**Hip adduction/abduction**				
maximum	-2.4	3.4	-9.2	4.3
minimum	2.1	5.0	-7.8	12.0
ROM	-4.5	5.2	-14.8	5.7
initial contact	1.3	5.6	-9.7	12.4
**Hip int/external rotation**				
maximum	-18.9	8.8	-36.2	-1.7
minimum	-14.0	10.3	-34.1	6.1
ROM	-4.9	5.4	-15.4	5.6
initial contact	-17.5	10.7	-38.5	3.6

**Knee flexion/extension**				
maximum	-0.6	12.7	-25.4	24.2
minimum	-4.6	10.8	-25.7	16.5
ROM	4.0	17.9	-31.1	39.1
initial contact	-3.8	14.0	-31.2	23.6
**Ankle dorsi/plantar flexion**				
maximum	-16.7	7.4	-31.2	-2.2
minimum	-5.2	8.0	-20.9	10.6
ROM	-11.5	5.8	-22.9	-0.1
initial contact	-3.1	7.8	-18.4	12.2

Table S3. Results of Bland-Altman analysis of agreement between joint kinematic parameter calculated from Kinect (calibrated by long short-term memory recurrent neural network) and Motion Analysis. Mean difference, limits of agreement (LoA), Lower LoA and Upper LoA are reported.

Kinematic parameter	Mean difference	LoA	Lower LoA	Upper LoA
**Hip flexion/extension**				
maximum	-3.1	10.1	-22.8	16.7
minimum	5.9	13.0	-19.6	31.5
ROM	-9.0	9.6	-27.9	9.9
initial contact	-1.6	9.5	-20.3	17.0
**Hip adduction/abduction**				
maximum	0.4	5.4	-10.2	11.0
minimum	0.5	4.9	-9.1	10.1
ROM	-0.1	7.1	-14.1	13.9
initial contact	0.4	6.3	-12.0	12.8
**Hip int/external rotation**				
maximum	0.9	12.2	-22.9	24.8
minimum	-1.4	11.0	-23.0	20.2
ROM	2.3	7.3	-12.0	16.6
initial contact	-0.1	11.6	-22.9	22.7
**Knee flexion/extension**				
maximum	-5.1	9.0	-22.8	12.5
minimum	1.6	11.8	-21.6	24.8
ROM	-6.7	12.8	-31.8	18.4
initial contact	-0.7	8.2	-16.8	15.3
**Ankle dorsi/plantar flexion**				
maximum	-0.6	7.8	-16.0	14.7
minimum	-0.8	8.2	-16.9	15.3
ROM	0.2	5.3	-10.3	10.6
initial contact	-1.2	7.8	-16.5	14.1
